# Effects of Low-Ambient-Temperature Stimulation on Modifying the Intestinal Structure and Function of Different Pig Breeds

**DOI:** 10.3390/ani12202740

**Published:** 2022-10-12

**Authors:** Yanbo Guo, Ting Liu, Wenxia Li, Wanfeng Zhang, Chunbo Cai, Chang Lu, Pengfei Gao, Guoqing Cao, Bugao Li, Xiaohong Guo, Yang Yang

**Affiliations:** College of Animal Science, Shanxi Agricultural University, No. 1 of South Mingxian Road, Jinzhong 030801, China

**Keywords:** low-ambient-temperature stimulation, intestinal structure, tight junction, inflammatory response, digestive enzyme activity

## Abstract

**Simple Summary:**

Low ambient temperature resulted in the body’s cold stress response, while local wild boars in the middle-temperate zone performed better than commercial pigs. Therefore, three breeds—Large White (LW) pigs, a local Mashen (MS) pig breed and Jinfen White (JFW) pigs, a hybrid breed from wild boar—were investigated in an artificial climate chamber. The results implicated that low-ambient-temperature stimulation increased trypsin activity in duodenal chyme and promoted inflammatory response in Mashen pigs. The cold-resistance mechanism of MS pigs should be explored to reduce hogs’ stress caused by low-ambient-temperature stimulation.

**Abstract:**

Ambient temperature (Ta) fluctuation is a key factor affecting the growth performance and economic returns of pigs. However, whether the response of intestinal structure and function are related to pig breeds in low Ta has not been investigated yet. In this study, Large White (LW) pigs, Jinfen White (JFW) pigs and Mashen (MS) pigs were raised in artificial climate chambers under normal Ta (25 °C) and low Ta (4 °C) for 96 h. Afterwards, the decrease in body temperature and complete blood counts (CBC) of all pigs were measured. Hematoxylin–eosin, immunohistochemical staining, qPCR and ELISA were used to investigate their intestinal mucosa integrity and inflammatory response. The results showed that MS pigs could maintain a normal body temperature and villus structure after 4 °C stimulation compared with those of LW and JFW pigs. Villus height and villus height/crypt depth of MS pigs were significantly higher than those of LW and JFW pigs at 4 °C. Low-Ta stimulation increased the digestion of carbohydrates of all pigs. Meanwhile, low Ta enhanced the activity of lipase in LW pigs and increased trypsin activity in MS and JFW pigs. Furthermore, low-Ta stimulation significantly downregulated the protein of tight junction and upregulated the mRNA expression of inflammatory cytokines in MS pigs. MS pigs also showed stronger spleen immune function at 4 °C. These results indicated that the local MS pig breed had stronger intestinal function in low Ta by producing a stronger inflammatory response, which lays the foundation for further study on the mechanism of cold tolerance in pigs.

## 1. Introduction

Low-ambient-temperature (Ta) exposure is a strong physiological environmental stressor. It is an important factor in shaping phenotypic plasticity, which could result in the body’s cold stress response involving changes in hormones [1,2,3], cardiovascular [4], nervous, muscular [5], immune systems [1,2,5,6,7] and the gastrointestinal tract [8]. As the organ with the largest contact area between the body and the external environment, the intestine is sensitive to Ta. In pigs, the intestine has a stronger intestinal mucosa immune response [9] and histological structure disorganization [10,11] when Ta decreases. Based on previous research, low Ta would also enlarge the villus length of the duodenum [12] and change the gut microflora [13]. Thus, considerable attention has been given to the effects of different low Ta on the intestinal barrier to understand the potential role of the intestinal tract in building cold resistance and improving productivity of livestock animals. Researchers have observed that low Ta would cause cecal villus atrophy and rupture and reduce the gastric absorption of nutrients [14,15]. Moreover, low Ta would modulate the secretion and release of inflammatory cytokines, and appropriate low-Ta stimulation could improve the immune function and ability of the small intestine to withstand cold stress and disease [7]. However, the mechanism by which temperature affects the phenotypic and morphological changes of the duodenum remains poorly understood.

In general, pigs with different genetic backgrounds have different cold-tolerance characteristics [16]. Wild boar distributed in northern China, such as Mashen pigs and Min pigs, has evolved strong cold-resistance features because of natural windy conditions and long-term random feeding methods. Regular exposure to a cold factor could increase tolerance to low ambient temperature because of numerous adaptive mechanisms [17]. Consequently, they have a low frostbite rate, low shivering rate, long shivering interval and low proportion of crowd lying. They can grow and develop normally outdoors in winter or under poor enclosure insulation compared with commercial pigs [18]. However, mechanisms underlying cold resistance of native wild boar remain unclear. This study selected the Mashen (MS) pig and its breed Jinfen White (JFW) pig as experimental material to explore the cold-resistance mechanism of local pig breeds.

MS pig is an excellent indigenous breed in Shanxi Province that is characterized by high fertility, good meat quality, strong resistance to stress and slow growth rate [19,20,21]. JFW pig is a local hybrid breed of multiple pigs, including Mashen (6.25%), Taihu (3.13%), Landrace (40.62%) and Yorkshire pigs (50%) [22]. They also have good-quality meat and strong stress resistance to MS pigs, but their growth rate is significantly superior to that of MS pigs [23].

In this study, Large White (LW) pigs, JFW pigs and MS pigs were used to reveal differences in cold tolerance of different pig breeds, which we hoped to find it through differences in digestive function, intestinal structure and inflammatory reaction. This study will investigate the cold-resistance mechanism of local pig breeds from the perspective of duodenal function and provide a reference for the study of cold resistance of different pig breeds and healthy breeding in winter.

## 2. Materials and Methods

In this study, pigs were provided by the Datong breeding pig farm (Datong, China). A total of 18 pigs were selected (6 in each breed). All pigs were healthy and similar in weight (30 ± 5 kg). Each breed was divided into the low-Ta group and the normal-Ta group so that there were three pigs in each group (*n* = 3). All animals were habituated in an artificial climate chamber at least 4 weeks before the experiments and offered commercial standard diets (Guangtai, Shanxi, China) and water *ad libitum*. During the experiment, pigs in the low-Ta group were maintained at 4 °C ± 1 °C for 96 h, whereas the normal-Ta group was maintained at 25 °C ± 1 °C as control. Each artificial climate room was set with a 12 h:12 h day and night cycle.

After 96 h of low-Ta stimulation, we measured the core temperature of each pig and collected their blood into K_2_EDTA anticoagulation tubes from the precaval vein to evaluate the complete blood counts (CBC) in all research objects. All animals were anesthetized by electric shock and slaughtered immediately by cutting off the carotid artery. Then, the duodenum and spleen were removed using aseptic techniques intactly and immediately. Two centimeters of intestinal and spleen segments were cut and washed with pre-cooling physiological saline and fixed in 4% paraformaldehyde solution for 72 h. Duodenal contents and tissue were collected and subpacked in a cryopreservation tube to quickly freeze in liquid nitrogen for 3 h and were then transferred to a refrigerator at −80 °C for subsequent testing.

All K_2_EDTA samples were measured based on CBC parameters using hematology analyzers (Mindray, Shenzhen, China), including red blood cell count (RBC), hemoglobin (HGB), red blood cell specific volume (HCT), platelet count (PLT), white blood cell count (WBC), lymphocyte rate (LYM%), intermediate cell rate (MID) and granulocyte rate (GR%).

The duodenal and spleen segments fixed in paraformaldehyde were trimmed and rinsed gently with running water for 16 h and then embedded in paraffin after decreasing the concentration of ethanol solution treatment by using standard methods. Paraffin sections with a thickness of 7 μm were cut out from each paraffin block, stuck to a slide, dried at 37 °C, and then stained with hematoxylin–eosin (H&E) staining for morphometric analysis. All specimens were evaluated under a 40× light microscope (Leica, Wetzlar, Germany) and photographed by using a digital camera (Leica, Wetzlar, Germany) through Image Pro Plus 6.0 (IPP) for histometric analyses. IPP was also used to measure the villus height, villus width and crypt depth of the gut mucosa. Measurements were made on five well-orientated villi randomly selected from different parts of five serial sections of each pig. Villus height was measured from the tip to the base of the villi. Villus width was obtained at sites where it was uniform [24], and crypt depth was measured from the submucosa to the villus base.

The expression of the tight junction (anti-zonula occludens 1 (*ZO-1*) antibodies, bs1329R; anti-occludin antibodies, bs10011R; Bioss, Beijing, China) was detected in duodenal tissue by immunohistochemistry (IHC) to evaluate the integrity of the intestinal mucosa barrier. Paraffin sections with a thickness of 3 μm were dewaxed in dimethylbenzene and rehydrated in different concentrations of gradient ethanol. After endogenous peroxidase was removed with a 3% H_2_O_2_ solution, the specimens were soaked in sodium citrate to repair their antigens by microwave. Bovine serum albumin was used to block nonspecific antigen sites at 37 °C. Tissue sections were incubated with an antibody at 4 °C for 18 h, and then goat anti-rabbit IgG and strept avidin-biotin complex (SABC) were incubated sequentially. 3,3N-Diaminobenzidine Tertrahydrochloride (DAB) reaction and hematoxylin staining were subsequently carried out under an optical microscope, followed by routine dehydration and clearing. Finally, all sections were mounted in a neutral balsam medium. *Occludin* and *ZO-1* were presented by histologic sections at 100× magnification. Image sequences were captured using a light microscope through IPP and quantified by calculating the percentage of DAB stain area through true-color image analysis using adjusted thresholds in ImageJ 1.8.0_172. Measurements were performed on five fields randomly selected from different parts of serial sections of each pig under a light microscope.

Total RNA was extracted from the duodenum tissue of pigs using TRIzol^®^ Reagent (Thermo Fisher, CA, USA). Its concentration and purity were determined spectrophotometrically at 260/280 nm and diluted to 500 ng/μL. First-strand cDNA was synthesized from 100 ng of total RNA following kit instructions (TransGen, Beijing, China). Subsequent qRT-PCR was used to detect the expression of the tight junction—*Occludin*, *ZO-1*—and inflammatory cytokines—*tumor necrosis factor alpha* (*TNF-α*), *interleukin 1 beta* (*IL-1β*), *IL-4*, *IL-6*, *IL-10*, *IL-15*. When primers (Table 1) and all solutions (TransGen, Beijing, China) were added to a 96-well plate according to the manufacturer’s protocol, real-time PCR was conducted on a CFX Connect real-time RT-PCR system (BioRad, Hercules, CA, USA) in accordance with the following steps: at 95 °C for 45 s, followed by 45 cycles of 95 °C for 7 s and 60 °C for 34 s, and a final cooling step at 40 °C for 60 s. Relative mRNA expression was normalized to 18S using the 2−ΔΔCt method [25]. Three independent RNA preparations were used as biological replicates, and their results were presented as mean ± SEM.

We determined the antibody concentration of tight junction proteins (*ZO-1*, *Occludin*) and inflammatory cytokines (*IL-4*, *IL-6*, *IL-10*, *TNF-α*) in intestine tissues using ELISA kits according to the manufacturer’s instructions (Mlbio, Shanghai, China). Intestinal tissue was rinsed with cold normal saline and made into 10% intestinal homogenate.

Duodenum tissue was homogenized in cold saline to prepare for the digestive enzyme activity assay. The activity of four enzymes—α-amylase, lipase, cellulase and trypsin—was determined by colorimetry using commercial kits (Jiancheng, Nanjing, China), and their code numbers are presented in Table S1.

Statistical analysis was conveyed as mean ± SEM and conducted using IBM SPSS Statistics 22.0. Differences between temperature, breeds and their interactions were evaluated by two-way analysis of variance (ANOVA) using GraphPad Prism (GraphPad Software, La Jolla, CA, USA). *p* < 0.05 was considered to indicate statistical significance.

## 3. Results

### 3.1. Body Temperature and Complete Blood Counts

**Figure 1:**
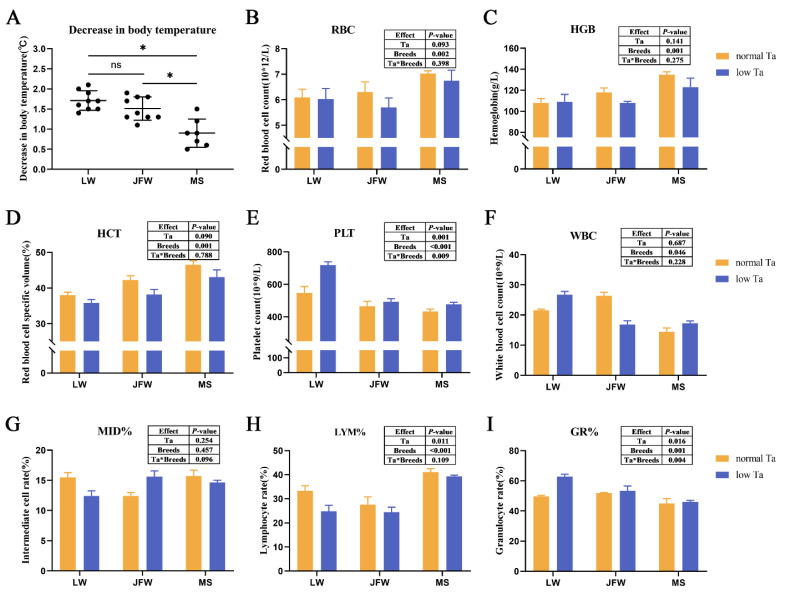
Effect of 96 h low-Ta stimulation on decrease in body temperature and complete blood counts (CBC) in three breeds of pigs. The difference of decrease in body temperature among three breeds was analyzed by one-way ANOVA. The effects of Ta and breeds and their interactions on CBC were analyzed by two-way ANOVA. (**A**) The decrease in body temperature (Black dots represent duplicate values in a group.); (**B**–**I**) complete blood counts. Abbreviation: Ta = ambient temperature; LW = Large White pigs; JFW = Jinfen White pigs; MS = Mashen pigs; RBC = red blood cell count; HGB = hemoglobin; HCT = red blood cell specific volume; PLT = platelet count; WBC = white blood cell count; LYM% = lymphocyte rate; MID = intermediate cell rate; GR% = granulocyte rate. Values show the means ± SEM (*n* = 3 × 3). ns: no significance. *: significant difference (*p* < 0.05).

Three breeds of pigs were acclimated to 25 °C (normal Ta) and 4 °C (low Ta) conditions. At the end of experiment, the decrease in body temperature of LW pigs (average 1.7 °C) had no disparity with that of JFW pigs (average 1.5 °C), and it was higher than that of MS pigs (average 0.9 °C, Figure 1A, Table S2) (*p* < 0.05). LYM% (*p* = 0.011), GR% (*p* = 0.016) and PLT (*p* = 0.001) were significantly affected by Ta (Figure 1B–I). Low-Ta stimulation diminished LYM% and increased GR% and PLT of all pigs significantly. RBC (*p* = 0.002), HGB (*p* = 0.001), HCT (*p* = 0.001), PLT (*p* < 0.001), WBC (*p* = 0.046), LYM% (*p* < 0.001) and GR% (*p* = 0.001) were significantly affected by breeds. LYM%, RBC, HGB and HCT of MS pigs were the highest among three breeds in normal and low Ta. GR% (*p* = 0.004) and PLT (*p* < 0.009) were significantly affected by the interaction between Ta and breeds, while MID% had a tendency of response to their interaction (*p* = 0.096). Additionally, the main effect of Ta had a tendency to influence RBC (*p* = 0.093) and HCT (*p* = 0.090).

### 3.2. Duodenal Pathological Observations

**Figure 2:**
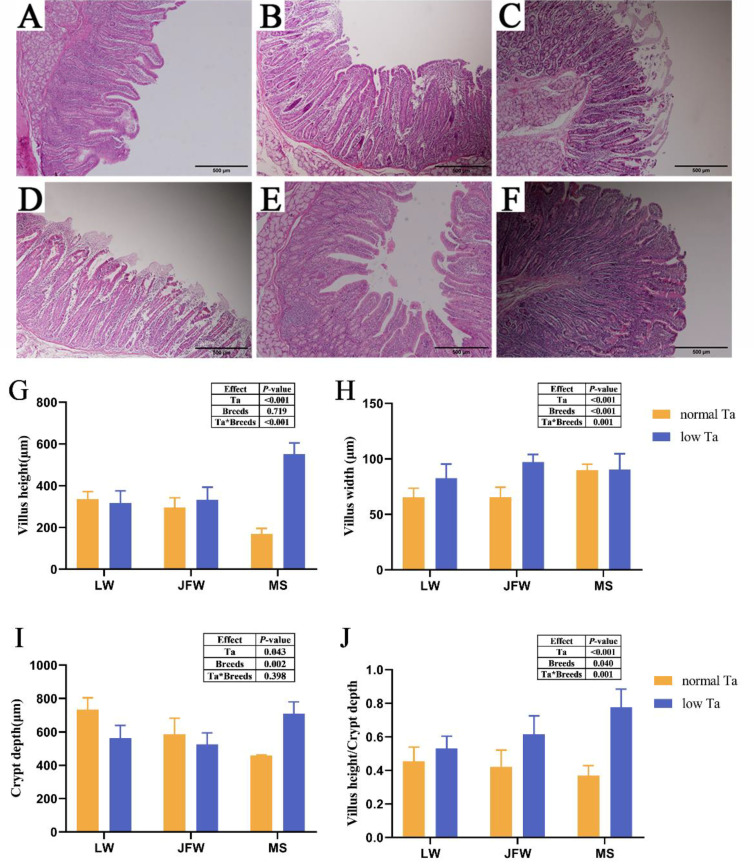
Effect of low-Ta stimulation on the histological morphology of the duodenum in three breeds of pigs. (**A**–**C**) H&E staining of the duodenum of three breeds exposed to normal Ta (40×): (**A**) H&E staining of the duodenum of LW pigs; (**B**) H&E staining of the duodenum of JFW pigs; (**C**) H&E staining of the duodenum of MS pigs. (**D**–**F**) H&E staining of the duodenum of three breeds restricted to low temperature (40×): (**D**) H&E staining of the duodenum of LW pigs; (**E**) H&E staining of the duodenum of JFW pigs; (**F**) H&E staining of the duodenum of MS pigs. (**G**–**J**) Morphometric quantifications of duodenal villi, the effects of Ta and breeds and their interactions were analyzed by two-way ANOVA. (**G**) Morphometric quantifications of villus height; (**H**) morphometric quantifications of villus width; (**I**) morphometric quantifications of crypt depth; (**J**) morphometric quantifications of villus height/crypt depth. *n* = 3 × 3, scale bar = 500 µm. The data in (**G**–**J**) show the means ± SEM.

Duodenal histology-manifested structures of all pigs in the normal-Ta group (Figure 2A–C) were clear and intact in the mucosa, whereas they were disordered, shrunk, and even fell off slightly in the low-Ta group (Figure 2D–F). LW pigs were more severely affected by low-Ta stimulation compared with JFW and MS pigs. We measured villus height, villus width and crypt depth to observe alterations in duodenal morphology intuitively (Figure 2G–J). The results revealed significant main effects of Ta in duodenal morphometric quantifications. Villus width (*p* < 0.001), crypt depth (*p* = 0.002) and villus height/crypt depth (*p* = 0.040) were significantly affected by breeds. It showed significant interactions effects of Ta and breeds for villus height, villus width and villus height/crypt depth. In normal Ta, LW pigs had the maximum villus height and crypt depth comparison with other breeds, and MS pigs had the minimum. Villus height, crypt depth and villus height/crypt depth of MS pigs had grown with low-Ta acclimation, and they were significantly superior to those of LW and JFW in low Ta. Villus width had no transformation during low-Ta stimulation in MS pigs.

### 3.3. Changes in Digestive Enzyme Activity

**Figure 3:**
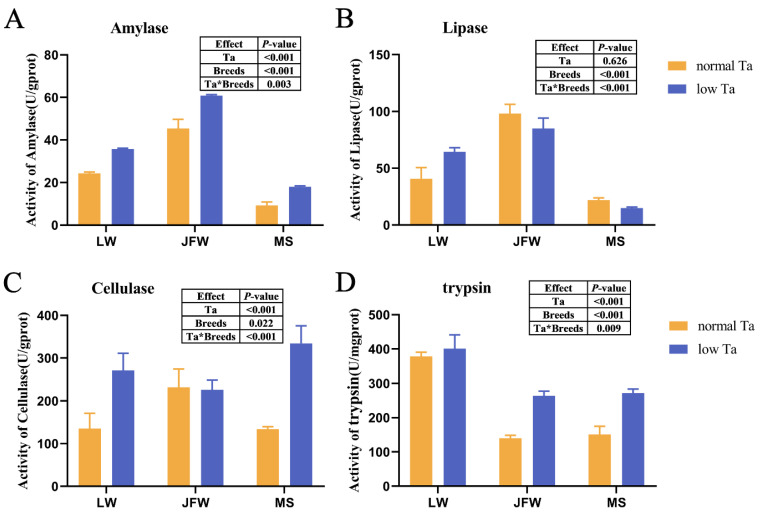
Digestive enzyme activity in three breeds at 25 °C and 4 °C. The effects of Ta and breeds and their interactions were analyzed by two-way ANOVA. (**A**) Activity of α-amylase; (**B**) activity of lipase; (**C**) activity of cellulase; (**D**) activity of trypsin. *n* = 3 × 3, data show the means ± SEM.

After low-Ta treatment, the activity of four digestive enzymes was determined. Trypsin activity of LW pigs was the highest among the three breeds in normal Ta (Figure 3), while α-amylase, lipase and cellulase activity of JFW pigs was the highest at 25 °C. The activity of most digestive enzymes in low Ta exceeded that in normal Ta: α-amylase, lipase and cellulase activity in LW pigs amplified, whereas α-amylase and trypsin activity in JFW pigs and α-amylase, cellulase and trypsin activity in MS pigs augmented prominently. However, lipase activity of MS pigs diminished at 4 °C. These data indicated that Ta treatment and breeds had an interaction effect on the activity of four digestive enzymes.

### 3.4. Tight Junction Expression

**Figure 4:**
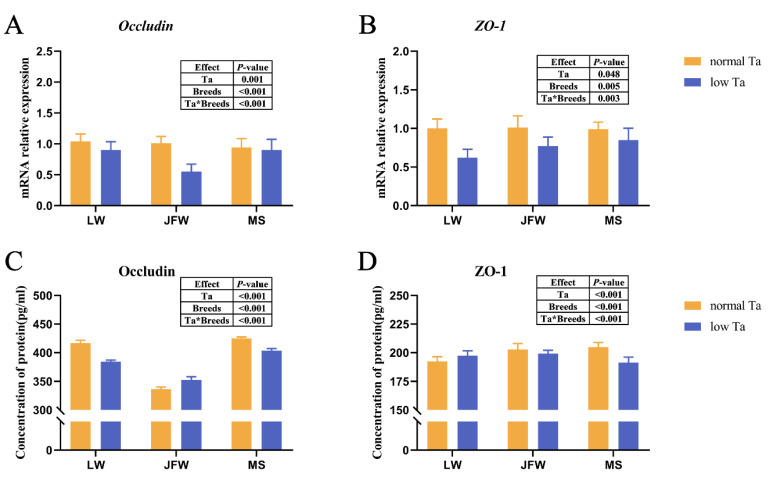
Tight junction of the duodenum in response to low-Ta stimulation in three breeds of pigs. The effects of Ta and breeds and their interactions were analyzed by two-way ANOVA. (**A**) The relative mRNA expression of *Occludin* in duodenum of three breeds; (**B**) the relative mRNA expression of *ZO-1* in duodenum of three breeds; (**C**) concentration of *Occludin* in duodenum of three breeds; (**D**) concentration of *ZO-1* in duodenum of three breeds. Abbreviation: *ZO-1* = zonula occludens 1.

The expression of tight junctions was significantly responsive to Ta and breeds and their interactions. Low-Ta exposure diminished the mRNA expression of *Occludin* and *ZO-1* in the duodenum of all pigs remarkably (Figure 4A,B). At 25 °C, MS pigs had the highest *Occludin* and *ZO-1* concentrations compared with those of other breeds (Figure 4C,D). Low-Ta stimulation decreased the concentration of *Occludin* and *ZO-1* in MS pigs and the concentration of *Occludin* in LW pigs. At 4 °C, MS pigs kept the highest concentration of *Occludin*, while JFW pigs had the highest *ZO-1* protein concentration.

**Figure 5:**
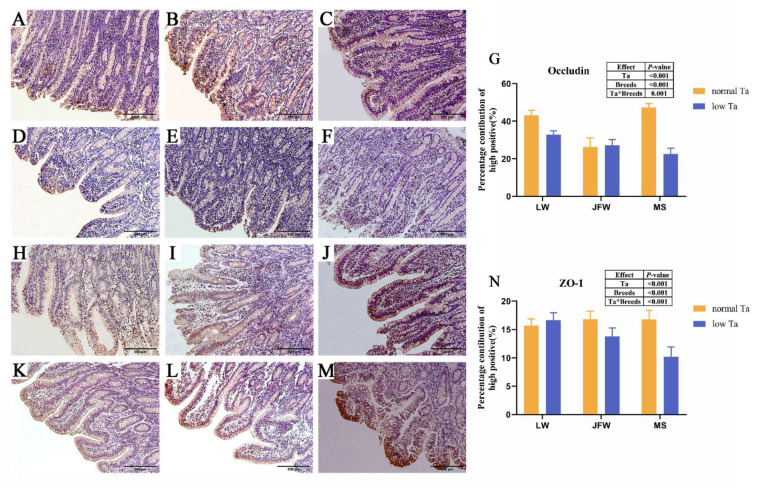
Effects of Ta stimulation on duodenal tight junction in three breeds of pigs (immunohistochemical (IHC) stain) and morphometric quantifications of the percentage of IHC stain area. The effects of Ta and breeds and their interactions were analyzed by two-way ANOVA. (**A**) IHC stain of *Occludin* in LW pigs at 25 °C; (**B**) IHC stain of *Occludin* in JFW pigs at 25 °C; (**C**) IHC stain of *Occludin* in MS pigs at 25 °C; (**D**) IHC stain of *Occludin* in LW pigs at 4 °C; (**E**) IHC stain of *Occludin* in JFW pigs at 4 °C; (**F**) IHC stain of *Occludin* in MS pigs at 4 °C; (**G**) morphometric quantification of IHC stained area percentage of *Occludin* in three breeds; (**H**) IHC stain of *ZO-1* in LW pigs at 25 °C; (**I**) IHC stain of *ZO-1* in JFW pigs at 25 °C; (**J**) IHC stain of *ZO-1* in MS pigs at 25 °C; (**K**) IHC stain of *ZO-1* in LW pigs at 4 °C; (**L**) IHC stain of *ZO-1* in JFW pigs at 4 °C; (**M**) IHC stain of *ZO-1* in MS pigs at 4 °C; (**N**) morphometric quantification of IHC stained area percentage of *ZO-1* in three breeds. *n* = 3 × 3, 100 ×; scale bars, 500 μm. The data in **G**–**J** show the means ± SEM.

The brown area of duodenal sections illustrated the location of *Occludin* (Figure 5A–F and Figure S1A–F) and *ZO-1* protein (Figure 5H–M and Figure S1G–L). The percentage contribution of positive sites of tight junctions were significantly responsive to Ta (*p* < 0.001), breeds (*p* < 0.001) and the interactions between Ta and breeds (*p* < 0.001). Low-Ta stimulation narrowed the percentage contribution of positive sites of *Occludin* protein in LW pigs and MS pigs (Figure 5G,N). In the low-Ta group, the percentage contribution of positive sites of *Occludin* and *ZO-1* in LW pigs was higher than that in MS and JFW pigs.

### 3.5. Inflammatory Cytokine Expression

**Figure 6:**
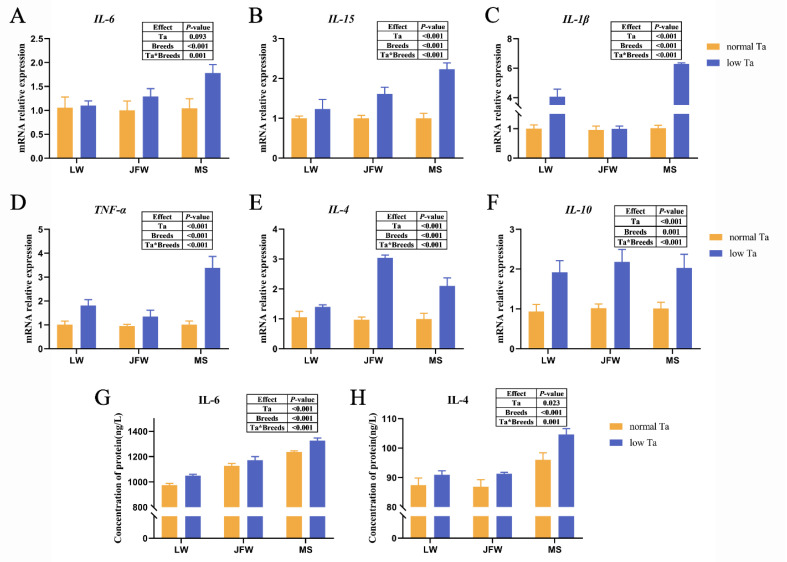
The expression of inflammatory cytokines in response to low-Ta stimulation in the duodenal mucosa of three pig breeds. The effects of Ta and breeds and their interactions were analyzed by two-way ANOVA. (**A**) Relative gene expression of *IL-6*; (**B**) relative gene expression of *IL-15*; (**C**) relative gene expression of *IL-1β*; (**D**) relative gene expression of *TNF-α*; (**E**) relative gene expression of *IL-4*; (**F**) relative gene expression of *IL-10*; (**G**) concentration of *IL-6*; (**H**) concentration of *IL-4*. Abbreviation: *IL-4* = interleukin 4; *IL-10* = interleukin 10; *IL-6* = interleukin 6; *IL-15* = interleukin 15; *IL-1β* = interleukin 1 beta; *TNF-α* = tumor necrosis factor alpha.

qPCR array analysis showed that the expression of cytokines was significantly affected by Ta and breeds and their interactions. The expression of *IL-4*, *IL-10*, *IL-15*, *IL-1β* and *TNF-α* in LW pigs of low-Ta group was elevated compared with those in the normal-Ta group (Figure 6A–F). Compared with the normal-Ta group, 96 h of low-Ta exposure significantly upregulated (*p* < 0.01) the mRNA expression level of *IL-4*, *IL-10*, *IL-6*, *IL-15* and *TNF-α* in JFW pigs. For MS pigs, the expression level of *IL-4*, *IL-10*, *IL-6*, *IL-15*, *IL-1β* and *TNF-α* mRNAs was significantly increased compared with that in normal Ta. At 4 °C, the expression level of pro-inflammatory cytokines in MS pigs and anti-inflammatory cytokines in JFW pigs was superior to that in other breeds. We also analyzed the concentration of some inflammatory cytokines by ELISA (Figure 6G,H). MS pigs possessed the highest level of *IL-6* and *IL-4* at 25 °C and 4 °C. The concentration of *IL-4* and *IL-6* in all breeds elevated with low-Ta stimulation.

### 3.6. Pathological Observation of Spleen

**Figure 7:**
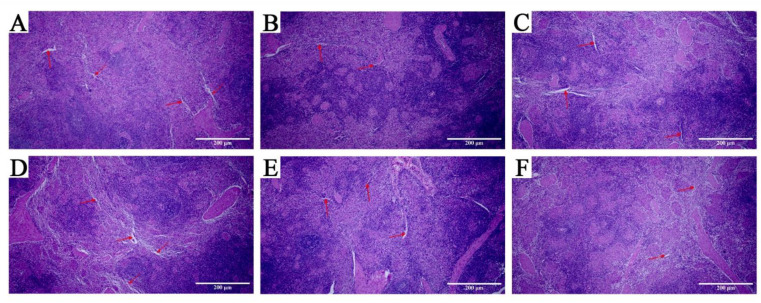
Effect of Ta stimulation on the spleen morphology of three pig breeds. (**A**) H&E stain of spleen in LW pigs at 25 °C; (**B**) H&E stain of spleen in JFW pigs at 25 °C; (**C**) H&E stain of spleen in MS pigs at 25 °C; (**D**) H&E stain of spleen in LW pigs at 4 °C; (**E**) H&E stain of spleen in JFW pigs at 4 °C; (**F**) H&E stain of spleen in MS pigs at 4 °C. *n* = 3 × 3, 40×; scale bars, 200 μm; the red arrow indicates the blood sinuses of the spleen.

A large number of blood sinuses appeared in LW pigs after 96 h of low-temperature stimulation (as indicated by the arrows in Figure 7) with a disordered structure, decreased lymphocytes, and unclear boundaries between white pulp and red pulp. In the low-Ta group, JFW pigs showed phenotypic changes similar to those in LW pigs. However, the number of blood sinuses in MS pigs dropped, and the boundary between white and red pulp could still be observed clearly and continuously without significant removal.

## 4. Discussion

Exposure to environmental stressors carries adverse consequences to bodies [26,27], reducing the growth performance of livestock and poultry and the income of animal husbandry. In general, local wild boar in middle-temperate zones of northern China has better performance than commercial pig does, which arises from genetic evolution and environmental acclimation. This study revealed the advantages of local pig breeds based on physical and intestinal functions.

The decrease in body temperature of MS pigs was lower than that of LW and JFW pigs, which indicated that MS pigs could adapt to low Ta and protect their body temperature from extreme low ambient temperature [17]. CBC was essential for assessing overall health. Related works report that lymphocyte and monocyte numbers diminish gradually and return to baseline during stress recovery [28]. In this study, LYM% of LW and JFW pigs decreased after 96 h of low-Ta stimulation, which was consistent with previous results. On the contrary, no change in LYM% was observed in MS pigs, which indicated that MS pigs would show more stable blood components when encountering low Ta.

As the “center of spiritual and physical strength” [29], the gastrointestinal tract influences many physiological processes, including digestion and mucosal immunity [13,30,31] through releasing several regulatory peptide hormones and regulating trafficking of macromolecules between the environment and the host through a barrier mechanism [32]. Digestive enzymes in the gastrointestinal tract are responsible for the hydrolysis of nutrients. Villi are distributed on the inner surface of the small intestinal wall to expand the contact area between the chyme and the intestinal wall and accelerate the absorption of nutrients. Villus height/crypt depth reflects the function of the intestine. Higher villus height/crypt depth denotes higher nutrient absorption and developmental capacity [33]. Our results showed that the villus height/crypt depth and digestive enzyme activity of MS pigs were lower than those of LW pigs at 25 °C, which indicates that the local wild boar has weak digestion and slow growth [23]. After low-Ta stimulation, villus height/crypt depth was significantly responsive to Ta and breeds and the interactions. Villus height/crypt depth of MS and JFW pigs was higher than that of LW pigs, which indicated that MS and JFW pigs had stronger absorption capacity in response to low Ta. The activity of α-amylase, cellulase and lipase in LW pigs augmented, whereas the activity of α-amylase, cellulase and trypsin in MS and JFW pigs increased. These results indicated that low-Ta stimulation could ameliorate the absorption rate of the intestine by changing the activity of digestive enzymes and promote the digestion of chyme to assist thermogenesis. LW pigs rely on the hydrolysis of carbohydrate and fat nutrients for energy at low Ta, whereas MS and JFW pigs depend on carbohydrate and protein nutrients primarily at 4 °C. Therefore, we hope to further investigate their cold-resistance phenotype from lipid metabolism.

Low-Ta exposure induces villi to swell, damage, atrophy, rupture and weaken the digestive capacity of the gastrointestinal tract in birds [14,15] and rats [34]. Stress exposure can cause the formation of gastric lesions [14]. Cecal tissues exposed to cold stress illustrate mucosal layer lesions, intestinal villus rupture and morphological damage [15]. Our results of H&E staining showed that the duodenal villi of pigs were ruptured and disorganized after low-Ta treatment. Villus rupture indicated the downregulation of tight junction genes such as *Occludin* and *ZO-1* that connect epithelial cells to control the permeability of the paracellular transport pathway and partly maintain the intestinal barrier function [35]. In this study, low-Ta treatment reduced the mRNAs expression and protein levels of the tight junction in MS pigs but increased the protein level of *ZO-1* in LW pigs. Immunohistochemical analysis also indicated that protein levels of the tight junction were decreased in MS pigs at low Ta. These data implied that the tight junctions of the duodenal mucosa in MS pigs were destroyed at 4 °C. However, the opposite changes of *Occludin* and *ZO-1* in LW pigs may indicate that duodenal mucosal damage in LW pigs was slighter than that in MS pigs. Moreover, the attenuation of the tight junction could rarefy the mucosa, which may lead to gut mucosal barrier dysfunction [36] and further cause diarrhea [37]. However, our pigs did not develop diarrhea.

Inflammatory cytokines play an important role in intestinal mucosal immunity [38,39], and their dynamic regulation may develop inflammation [40]. Based on previous studies, low-Ta stimulation could increase the level of proinflammatory cytokines [41]. In this study, 96 h of low Ta significantly upregulated the expression levels of inflammatory cytokines, which indicated that the body may alleviate the stress response by adjusting the expression level of inflammatory cytokines apace after low-Ta stimulation. MS pigs possessed higher pro-inflammatory cytokines than LW pigs did after 96 h of low-Ta stimulation, suggesting that MS pigs may be induced to produce stronger inflammatory response under low-temperature stimulation, which overturned our expectations. Furthermore, normal spleen morphology in low-Ta conditions implied that the immune response of MS pigs was more intense than that of LW and JFW pigs. Cytokines are key mediators of cellular interactions in the intestine. ELISA showed that the concentration of inflammatory cytokines in MS pigs was the highest compared to that in other breeds. According to previous reports, the secretion of inflammatory cytokines could reduce damage caused by cold stress [42]. These results suggest that the duodenal tight junction of MS pigs will be damaged at low Ta, and the immune function will be improved to adapt to low Ta by increasing the secretion of inflammatory cytokines in MS pigs. The mechanism of cold resistance of Mashen pigs needs to be further explored.

## 5. Conclusions

A local Mashen pig breed had a stronger ability to maintain the physiological function of the intestine compared with that of Large White pigs exposed to extreme low ambient temperature. It indicated that Mashen pigs had better cold-tolerance characteristics than Large White pigs did. Considering that they are bred from Mashen pigs, Jinfen White pigs also had a cold-resistance characteristic to a certain extent. This study provides a reference for the cold-resistance phenotype of different pig breeds.

## Figures and Tables

**Table 1:** Gene-specific primers information for qPCR.

Genes	Primer Sequences (5′ -> 3′)	Product Length (bp)	GenBank Accession No.
*Occludin*	F: CGAGACAGACTACACGACGG R: TTCATCAGCAGCAGCCATGT	247	NM_001163647.2
*ZO-1* ^1^	F: AGCCCGAGGCGTGTTT R: GGTGGGAGGATGCTGTTG	147	XM_021098891.1
*IL-4* ^2^	F: TCACCTCCCAACTGATCCCA R: GCTCCATGCACGAGTTCTTT	144	NM_214123.1
*IL-10* ^3^	F: CCACAAGTCCGACTCAACGA R: GGCAACCCAGGTAACCCTTA	267	NM_214041.1
*IL-6* ^4^	F: AGACCCTGAGGCAAAAGGGAAA R: CGGCATCAATCTCAGGTGCC	209	NM_214399.1
*IL-15* ^5^	F: TGCATCCAGTGCTACTTGTGT R: CCTGCACTGATACAGCCCAA	92	NM_214390.1
*IL-1β* ^6^	F: CCAATTCAGGGACCCTACCC R: GTTTTGGGTGCAGCACTTCAT	174	NM_214055.1
*TNF-α* ^7^	F: TGCACTTCGAGGTTATCGGC R: CGGCTTTGACATTGGCTACAA	141	NM_214022.1

^1^*ZO-1* = zonula occludens 1; ^2^ *IL-4* = interleukin 4; ^3^ *IL-10* = interleukin 10; ^4^ *IL-6* = interleukin 6; ^5^ *IL-15* = interleukin 15; ^6^ *IL-1β* = interleukin 1 beta; ^7^ *TNF-α* = tumor necrosis factor alpha.

## Data Availability

None of the data were deposited in an official repository. The data are available from the authors upon request.

## Supplementary Materials

The following supporting information can be downloaded at: https://www.mdpi.com/article/10.3390/ani12202740/s1, Table S1: Code number and detection principle of the digestive enzyme activity test kit; Table S2: Body temperature of all pigs; Figure S1: The negative images of IHC (100×).
