# Supplementary material for: Effects of Low-Ambient-Temperature Stimulation on Modifying the Intestinal Structure and Function of Different Pig Breeds
Source: Animals (Basel). 2022 Oct 12;12(20):2740. doi: 10.3390/ani12202740 (PMC9597737; doi:10.3390/ani12202740)
Supplement: Supplementary file 1 [file animals-12-02740-s001.zip › animals-1861989-supplementary.pdf]

Table S1. Code number and detection principle of the digestive enzyme activity test kit.

Enzyme	Detection principle	Item no.	CV
α-Amylase	Starch-iodine colorimetry	C016-1-1	1.7%
Lipase	Colorimetry	A054-1-1	1.7%
Cellulase	Colorimetry	A138-1-1	1.7%
Trypsin	UV colorimetry	A080-2-2	1.7%

Table S2. Body temperature of all Pigs.

Breed	Group	Body temperature before low Ta stimulation	Body temperature after low Ta stimulation
LW	normal Ta [1]	39.56 ± 0.13	39.88 ± 0.47
	low Ta	39.62 ± 0.11	38.16 ± 0.31
JFW	normal Ta	39.77 ± 0.16	39.78 ± 0.19
	low Ta	39.65 ± 0.20	38.23 ± 0.24
MS	normal Ta	39.64 ± 0.25	39.05 ± 0.17
	low Ta	39.45 ± 0.13	38.16 ± 0.21

[1] Pigs in the normal Ta group were maintained at 25 °C ± 1 °C for 96 h. Whereas the low Ta group were maintained at 4 °C ± 1 °C for 96 h.

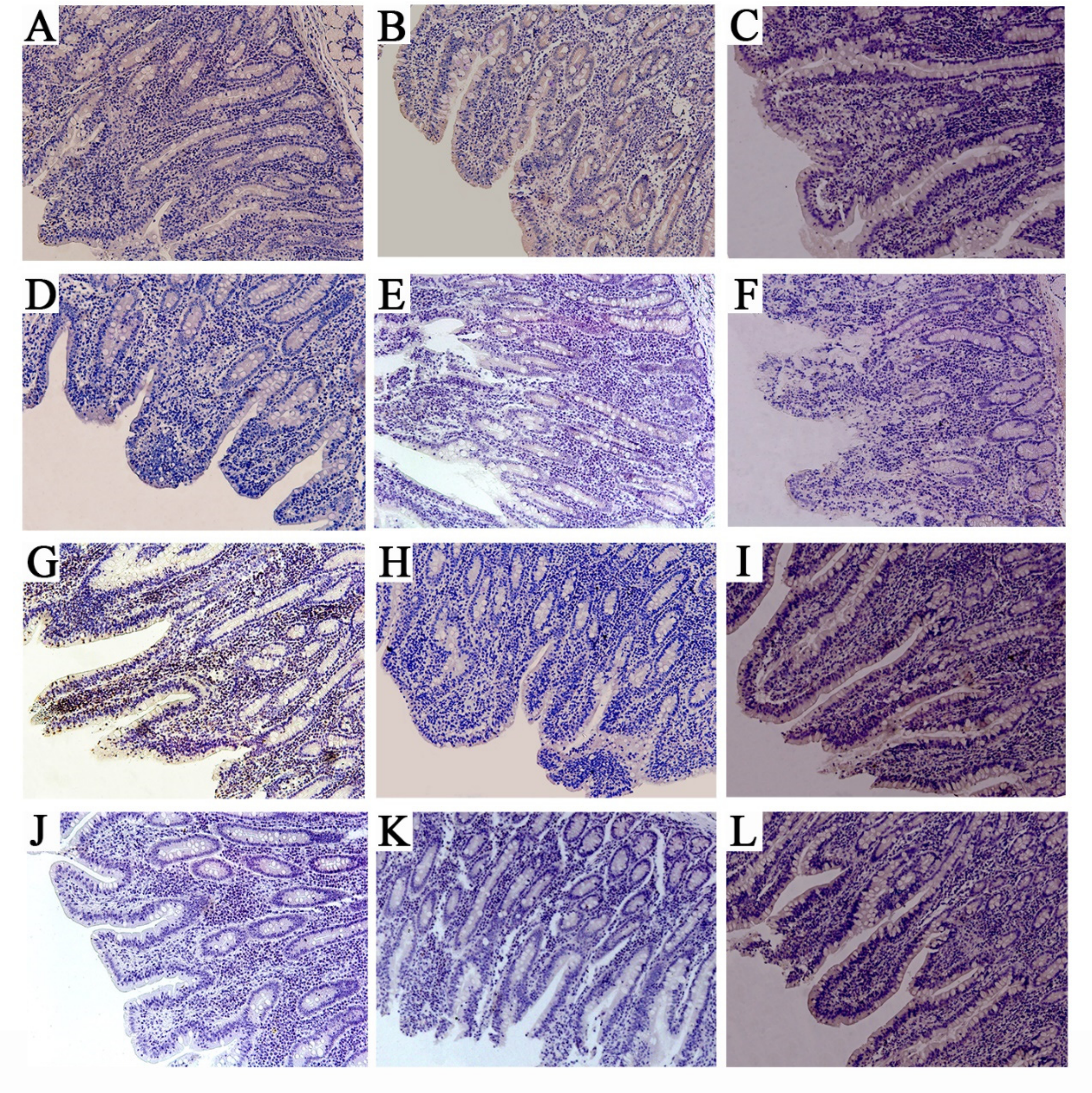

Figure S1. The negative images of IHC (100×). (**A**) Occludin negative of duodenum in LW pigs at 25 °C; (**B**) Occludin negative of duodenum in JFW pigs at 25 °C; (**C**) Occludin negative of duodenum in MS pigs at 25 °C; (**D**) Occludin negative of duodenum in LW pigs at 4 °C; (**E**) Occludin negative of duodenum in JFW pigs at 4 °C; (**F**) Occludin negative of duodenum in MS pigs at 4 °C; (**G**) ZO-1 negative of duodenum in LW pigs at 25 °C; (**H**) ZO-1 negative of duodenum in JFW pigs at 25 °C; (**I**) ZO-1 negative of duodenum in MS pigs at 25 °C; (**J**) ZO-1 negative of duodenum in LW pigs at 4 °C; (**K**) ZO-1 negative of duodenum in JFW pigs at 4 °C; (**L**) ZO-1 negative of duodenum in MS pigs at 4 °C.
